# Neighborhood disadvantage is associated with compromised white matter microstructure among older adults over a 9‐year follow‐up period

**DOI:** 10.1002/alz.095610

**Published:** 2025-01-09

**Authors:** Francis E. Cambronero, Panpan Zhang, Marissa A. Gogniat, Elizabeth E. Moore, Kimberly R. Pechman, L. Taylor Davis, Katherine A. Gifford, Bennett A. Landman, Dandan Liu, Timothy J. Hohman, Angela L. Jefferson

**Affiliations:** ^1^ Vanderbilt Memory & Alzheimer’s Center, Vanderbilt University Medical Center, Nashville, TN USA; ^2^ University of Pittsburgh Medical Center, Pittsburgh, PA USA; ^3^ Brigham and Women’s Hospital, Boston, MA USA; ^4^ Vanderbilt University, Nashville, TN USA

## Abstract

**Background:**

Socioeconomic neighborhood disadvantage has been linked to accelerated biological aging, cognitive decline, and core Alzheimer’s disease neuropathology independent of individual‐level factors. Our recent work indicates neighborhood disadvantage is also implicated in cerebrovascular changes known to exacerbate the development of AD, including neurovascular and hemodynamic dysfunction. Here, we investigated how neighborhood disadvantage relates to microstructural changes in white matter, a sensitive biomarker for emerging cerebrovascular disease and related neurodegeneration.

**Methods:**

Vanderbilt Memory and Aging Project participants (n = 316, 73±7 years, 41% female) underwent 3T multimodal MRI serially over a 9‐year follow‐up period (mean follow‐up = 5.8 years). Area Deprivation Index (ADI), representing neighborhood disadvantage, was quantified at baseline based on 17 components (e.g., income, education, employment, housing conditions). Diffusion tensor imaging (DTI) data were acquired and post‐processed through an established tract‐based spatial statistics pipeline to generate fractional anisotropy (FA), mean diffusivity (MD), radial diffusivity (RD), and axial diffusivity (AD) measures. Linear regressions related baseline ADI to each DTI measure adjusting for age, sex, race/ethnicity, education, cognitive status, Framingham Stroke Risk Profile (minus age), and apolipoprotein ε4 status. Linear mixed effects regression models related baseline ADI to longitudinal changes in each DTI measure adjusting additionally for follow‐up time.

**Results:**

In cross‐sectional analyses, higher baseline ADI was related to lower AD in the cingulum tract (β = ‐3.16^‐07, *p*‐value = 0.04) but unrelated to other tracts examined (*p*‐values>0.08). In longitudinal analyses, higher baseline ADI was related to increased AD (β = 1.241^‐7, *p*‐value = 0.04), MD (β = 1.193^‐7, *p*‐value = 0.04), and RD (β = 1.150^‐7, *p*‐value = 0.05) in the fornix tract over time. Baseline ADI was unrelated to longitudinal changes in other tracts examined (*p*‐values>0.20).

**Conclusions:**

Neighborhood disadvantage relates to worse white matter integrity among older adults at study entry and over time, particularly in the fornix and other limbic white matter tracts. This observation is particularly noteworthy given the modest level of disadvantage seen in this cohort. Findings support the hypothesis that adverse environmental conditions, including low‐quality housing and community‐wide resource constraints, may promote white matter tract degradation reflective of early vascular dysfunction or neurodegeneration. Future studies should investigate biological and behavioral correlates mediating observed associations, including the role of social stressors, built environment, and access to high quality health resources.

